# A80 DEVELOPMENT OF COMPETENCY BASED MEDICAL EDUCATION CURRICULUM FOR INFLAMMATORY BOWEL DISEASE ADVANCED TRAINING PROGRAM IN CANADA

**DOI:** 10.1093/jcag/gwac036.080

**Published:** 2023-03-07

**Authors:** R P Yanofsky, Z Gallinger

**Affiliations:** Gastroenterology & Hepatology, University of Toronto, Toronto, Canada

## Abstract

**Background:**

Inflammatory bowel disease (IBD) advanced training programs in Canada are heterogenous in their education, structure, and case exposure, resulting in differences in trainee experience. While competency based medical education (CBME) is becoming the primary educational framework in residency training, there is no CBME-based curriculum for IBD advanced training programs. Adopting a CBME framework for IBD fellowships may help standardize training to increase competence.

**Purpose:**

The purpose of this study was to develop a CBME-based curriculum for IBD advanced training programs in Canada.

**Method:**

Strategic groups consisting of 25 IBD experts were assigned to develop 15 entrustable professional activities (EPAs), which were then refined by experts in medical education and curriculum development. Acceptable EPAs were assessed using an electronically administered round-less Delphi panel. Panel members evaluated each EPA using a Likert scale, which asked participants to consider appropriateness of each EPA for an advanced IBD curriculum. A consensus strength greater than 70% was the acceptable threshold for inclusion into the final curriculum. Milestones were modelled after CanMED roles for each EPA.

**Result(s):**

Of the 15 initial EPAs, nine were recommended for the roundless Delphi panel by the selection committee: (1) identification, diagnosis, and treatment of new onset IBD; (2) assessment and treatment of outpatients with IBD; (3) the endoscopic evaluation of disease activity and the effective communication of endoscopic findings for patients with IBD; (4) provide effective inpatient management and coordinate transition to outpatient care for patients with IBD; (5) recognize and institute preventive health maintenance strategies in patients with IBD; (6) management and treatment of special populations with IBD; (7) identification, diagnosis, and treatment of the various extraintestinal manifestations of IBD; (8) Engage health care systems to improve the quality of care for patients with IBD; (9) engage in scholarly activities related to IBD. Eight of the nine EPAs achieved a consensus score greater than 70%, with the EPA, ‘engage in scholarly activities related to IBD’ failing to meet this cut-off. Associated milestones were developed for the eight final EPAs.

**Image:**

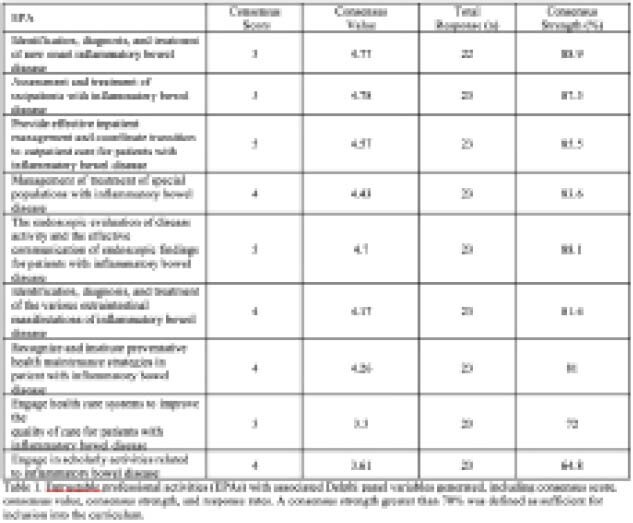

**Conclusion(s):**

This study was the first attempt to develop a CBME-based IBD curriculum in Canada. With proper funding and guidance, we hope our model can be implemented across Canada in IBD advanced training programs. These methods may also help facilitate the development of CBME curricula for other advanced training programs.

**Please acknowledge all funding agencies by checking the applicable boxes below:**

None

**Disclosure of Interest:**

None Declared

